# Aqua­(azido)[*N*-(pyridin-2-ylcarbon­yl)pyridine-2-carboxamido-κ^3^ *N*,*N*′,*N*′′]copper(II)

**DOI:** 10.1107/S1600536813027499

**Published:** 2013-10-12

**Authors:** Sandra Bruda, Mark M. Turnbull, Jan L. Wikaira

**Affiliations:** a Department of Chemistry, Clark University, 950 Main St, Worcester, MA 01610, USA; b Department of Chemistry, University of Canterbury, Private Bag 4800, Christchurch, New Zealand

## Abstract

The title compound, [Cu(C_12_H_8_N_3_O_2_)(N_3_)(H_2_O)], was formed by the air oxidation of 2-(amino­meth­yl)pyridine in 95% ethanol in the presence of copper(II) nitrate and sodium azide with condensation of the resulting picolinamide mol­ecules to generate the imide moiety. The Cu^II^ ion has a square-pyramidal coordination sphere, the basal plane being occupied by four N atoms [two pyridine (py) N atoms, the imide N atom and an azide N atom] in a nearly planar array [mean deviation = 0.048 (6) Å] with the Cu^II^ ion displaced slightly from the plane [0.167 (5) Å] toward the fifth ligand. The apical position is occupied by a coordinating water mol­ecule [Cu—O = 2.319 (4) Å]. The crystal structure is stabilized by hydrogen-bonding inter­actions between the water mol­ecules and carbonyl O atoms. The inversion-related square-pyramidal complex molecules pack base-to-base with long Cu⋯N_py_ contact distances of 3.537 (9) Å, preventing coordination of a sixth ligand.

## Related literature

For magneto-structural relationships in Cu^II^ complexes, see: Landee & Turnbull (2013 ▶). For copper(II)-catalysed air-oxidation of 2-amino­methyl­pyridine, see: Sahu *et al.* (2010 ▶); Turnbull *et al.* (2013 ▶). For the corresponding dicyanamide complex, see: Vangdal *et al.* (2002 ▶) and for the tri­cyano­methanide complex, see: de Gomes *et al.* (2008 ▶). For the bromide complex, see: Zhou *et al.* (2006 ▶) and for the fluoride and formate analogues, see: Borras *et al.* (2007 ▶). For the cyanate and thio­cyanate complexes, see: Dey *et al.* (2002 ▶) and Madariaga *et al.* (1991 ▶), respectively. For a related 2-amino­methyl­pyridine structure, see: Bruda *et al.* (2006 ▶). For the τ parameter as a geometry predictor in coordination complexes, see: Addison *et al.* (1984 ▶).
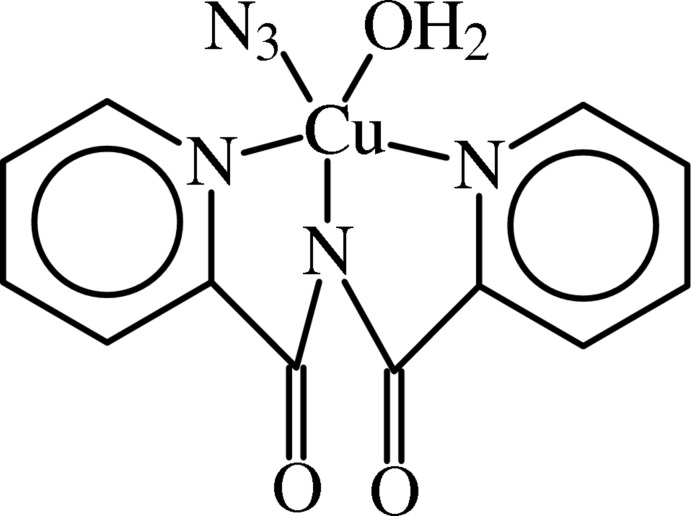


## Experimental

### 

#### Crystal data

[Cu(C_12_H_8_N_3_O_2_)(N_3_)(H_2_O)]
*M* *_r_* = 349.80
Triclinic, 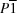

*a* = 7.402 (4) Å
*b* = 8.900 (5) Å
*c* = 10.606 (6) Å
α = 78.186 (9)°
β = 84.118 (8)°
γ = 70.040 (7)°
*V* = 642.4 (6) Å^3^
*Z* = 2
Mo *K*α radiation
μ = 1.72 mm^−1^
*T* = 168 K
0.28 × 0.06 × 0.03 mm

#### Data collection

Bruker SMART CCD area-detector diffractometer
Absorption correction: multi-scan (*SADABS*; Sheldrick, 1996 ▶) *T*_min_ = 0.854, *T*_max_ = 1.000
7577 measured reflections
2258 independent reflections
1837 reflections with *I* > 2σ(*I*)
*R*_int_ = 0.063

#### Refinement

*R*[*F* ^2^ > 2σ(*F* ^2^)] = 0.063
*wR*(*F* ^2^) = 0.105
*S* = 1.17
2258 reflections
205 parameters
2 restraints
H atoms treated by a mixture of independent and constrained refinement
Δρ_max_ = 0.50 e Å^−3^
Δρ_min_ = −0.69 e Å^−3^

### 

Data collection: *SMART* (Siemens, 1996 ▶); cell refinement: *SAINT* (Siemens, 1996 ▶); data reduction: *SAINT*; program(s) used to solve structure: *SHELXS97* (Sheldrick, 2008 ▶); program(s) used to refine structure: *SHELXL97* (Sheldrick, 2008 ▶); molecular graphics: *SHELXTL* (Sheldrick, 2008 ▶); software used to prepare material for publication: *SHELXL97*, *enCIFer* (Allen *et al.*, 2004 ▶) and *publCIF* (Westrip, 2010 ▶).

## Supplementary Material

Crystal structure: contains datablock(s) I, New_Global_Publ_Block. DOI: 10.1107/S1600536813027499/sj5358sup1.cif

Structure factors: contains datablock(s) I. DOI: 10.1107/S1600536813027499/sj5358Isup2.hkl

Additional supplementary materials:  crystallographic information; 3D view; checkCIF report

## Figures and Tables

**Table 1:** Hydrogen-bond geometry (Å, °)

*D*—H⋯*A*	*D*—H	H⋯*A*	*D*⋯*A*	*D*—H⋯*A*
O1—H1*A*⋯O9^i^	0.85 (2)	2.10 (4)	2.843 (5)	145 (5)
O1—H1*B*⋯O7^ii^	0.84 (2)	2.13 (3)	2.922 (5)	157 (5)
O1—H1*A*⋯O7^i^	0.85 (2)	2.45 (4)	3.105 (5)	134 (5)

Symmetry codes: (i) 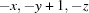; (ii) 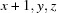.

**Fig. 1.**
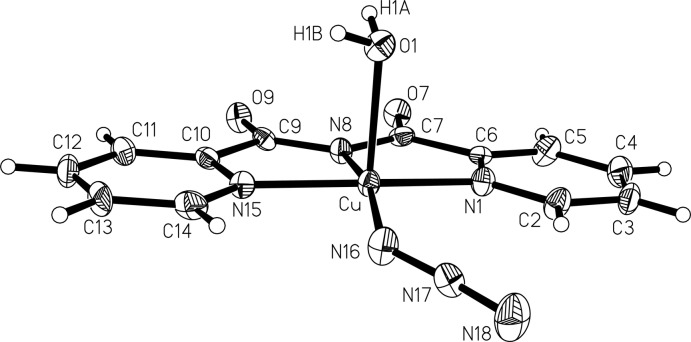
- Thermal ellipsoid plot (50%) of the molecular unit of (1). Only those H-atoms whose positions were refined are labeled.

**Fig. 2.**
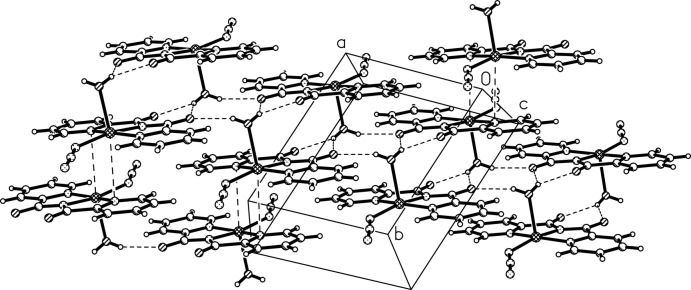
- Packing diagram of (1) showing hydrogen bonds and short Cu···N intermolecular contacts (dashed lines).

## supplementary crystallographic information

### 1. Comment

We are inter­ested in the design and synthesis of Cu^II^ complexes to study magnetostructural relationships in low-dimensional magnetic lattics (Landee and Turnbull, 2013). In this work, a wide variety of heterocyclic amines such as substituted pyrazine and pyridine compounds have been employed both as ligands and as bases. One such compound has been 2-amino­methyl­pyridine (Bruda *et al.*, 2006). We were attempting the preparation of a series of Cu^II^ complexes employing the 2-amino­methyl­pyridine molecule as a blocking agent to limit coordination by other species when we encountered the Cu^II^ catalyzed air-oxidation and condensation of 2-amino­methyl­pyridine and resulting *in situ* formation of a Cu^II^ nitrate complex of N-(pridin-2-ylcarboyl)pyridine-2-carbamide (bis-picolinimide; bpa) (Turnbull *et al.*, 2013). A similar reaction, in the presence of azide ion, has shown the same oxidation and condensation resulting in the preparation of [(N-(pyridin-2-ylcarboyl)pyridine2-carboxamido)(azido)(aqua)­copper(II)] (1).

Crystals of (1) (Figure 1) were produced via slow crystallization in air of an ethano­lic solution of Cu(NO_3_)_2_·3H_2_O, 2-amino­methyl­pyridine and sodium azide. The Cu^II^ ion is coordinated by one bpa anion, one azide anion and a water molecule to generate a nearly square pyramidal, five-coordinate structure. The basal plane is composed of three nitro­gen atoms from the bpa ligand and the coordinated azide ion. The four N-atoms are planar within 0.048 (6) Å and the Cu^II^ ion is displaced 0.167 (5) Å out of this plane. The O-atom of the water molecule is located in the apical position [Cu—O = 2.319 (4)Å]. The Addison parameter is 0.053, indicating that the geometry is very close to square pyramidal (Addison *et al.*, 1984).

The pyridine rings in the bpa ligand are virtually planar (mean deviation of constituent atoms = 0.0021 (8) N1-ring; 0.003 (2) N15-ring) and the rings themselves are nearly co-planar (2.5 (1)°). The lattice structure of (1) is supported by hydrogen bonds between the coordinated water molecule (donor) and the carbonyl oxygen atoms (acceptor) of adjacent bpa ligands (see Figure 2, Table 1). The five-coordinate nature of the Cu^II^ ion is stabilized by long inter­molecular Cu···N contacts between inversion related molecules [dCu···N15A = 3.537 (9) Å (-x, 2-y, -z)] effectively blocking the basal face of the square pyramidal structure and preventing coordination of a sixth ligand (see Figure 2).

Sahu and co-workers (Sahu *et al.*, 2010) have previously observed the copper catalyzed air-oxidation and condensation of 2-amino­methyl­pyridine to bpa as well as the corresponding reaction for 2-amino­methyl­quinoline. Similar structures have been reported with other inorganic anions replacing the azide ion including halides [Br, (Zhou *et al.*, 2006); F, (Borras *et al.*, 2007)], pseudo halides [OCN, (Dey *et al.*, 2002); SCN, (Madariaga, *et al.*, 1991)] and cyanamide derivatives (Vangdal, *et al.*, 2002; de Gomes *et al.* 2008)].

### 2. Experimental

Cu(NO_3_)_2_.3H_2_O, ethanol and NaN_3_ were obtained from VWR Scientific while 2-amino­methyl­pyridine was purchased from Aldrich Chemical. All were used as received.

### 2.1 Synthesis and crystallization

Cu(NO_3_)_2_^.^3H_2_O (0.241 g, 1.00 mmol), NaN_3_ (0.071g, 1.1 mmol) and 2-amino­methyl­pyridine (0.237 g,2.20 mmol) were dissolved in 20 ml of 95% ethanol in a 50 mL beaker and the beaker covered with parafilm with a couple of small holes in the film. Over the course of 2 weeks, blue needle-shaped crystals of (1) formed, which were isolated by filtration to give (1) 0.082 g (23 %).

### 2.2. Refinement

All H-atoms bound to carbon were placed in calculated positions (C—H = 0.95 Å) and refined using a riding model with *U*_iso_=1.2*U*_eq_ (C). Hydrogen atoms bonded to oxygen atoms were located in the difference map and their positions allowed to refine using anti-bumping restraints (0.85 Å for O—H distances) and fixed isotropic U values [*U*_iso_=1.2*U*_eq_ (O)].

### Crystal data

**Table:**

[Cu(C_12_H_8_N_3_O_2_)(N_3_)(H_2_O)]	*Z* = 2
*M**_r_* = 349.80	*F*(000) = 354
Triclinic, *P*1	*D*_x_ = 1.808 Mg m^−^^3^
Hall symbol: -P 1	Mo *K*α radiation, λ = 0.71073 Å
*a* = 7.402 (4) Å	Cell parameters from 2526 reflections
*b* = 8.900 (5) Å	θ = 2.9–26.0°
*c* = 10.606 (6) Å	µ = 1.72 mm^−^^1^
α = 78.186 (9)°	*T* = 168 K
β = 84.118 (8)°	Needle, blue
γ = 70.040 (7)°	0.28 × 0.06 × 0.03 mm
*V* = 642.4 (6) Å^3^	

### Data collection

**Table:**

Bruker SMART CCD area-detector diffractometer	2258 independent reflections
Radiation source: fine-focus sealed tube	1837 reflections with *I* > 2σ(*I*)
Graphite monochromator	*R*_int_ = 0.063
φ & ω scans	θ_max_ = 25.0°, θ_min_ = 2.5°
Absorption correction: multi-scan (*SADABS*; Sheldrick, 1996)	*h* = −8→8
*T*_min_ = 0.854, *T*_max_ = 1.000	*k* = −9→10
7577 measured reflections	*l* = −12→12

### Refinement

**Table:**

Refinement on *F*^2^	Primary atom site location: structure-invariant direct methods
Least-squares matrix: full	Secondary atom site location: difference Fourier map
*R*[*F*^2^ > 2σ(*F*^2^)] = 0.063	Hydrogen site location: inferred from neighbouring sites
*wR*(*F*^2^) = 0.105	H atoms treated by a mixture of independent and constrained refinement
*S* = 1.17	*w* = 1/[σ^2^(*F*_o_^2^) + (0.0191*P*)^2^ + 2.3778*P*] where *P* = (*F*_o_^2^ + 2*F*_c_^2^)/3
2258 reflections	(Δ/σ)_max_ = 0.001
205 parameters	Δρ_max_ = 0.50 e Å^−^^3^
2 restraints	Δρ_min_ = −0.69 e Å^−^^3^

### Special details

**Table:**

Geometry. All e.s.d.'s (except the e.s.d. in the dihedral angle between two l.s. planes) are estimated using the full covariance matrix. The cell e.s.d.'s are taken into account individually in the estimation of e.s.d.'s in distances, angles and torsion angles; correlations between e.s.d.'s in cell parameters are only used when they are defined by crystal symmetry. An approximate (isotropic) treatment of cell e.s.d.'s is used for estimating e.s.d.'s involving l.s. planes.
Refinement. Refinement of *F*^2^ against ALL reflections. The weighted *R*-factor *wR* and goodness of fit *S* are based on *F*^2^, conventional *R*-factors *R* are based on *F*, with *F* set to zero for negative *F*^2^. The threshold expression of *F*^2^ > σ(*F*^2^) is used only for calculating *R*-factors(gt) *etc*. and is not relevant to the choice of reflections for refinement. *R*-factors based on *F*^2^ are statistically about twice as large as those based on *F*, and *R*- factors based on ALL data will be even larger.

### Fractional atomic coordinates and isotropic or equivalent isotropic displacement parameters (Å^2^)

**Table:**

	*x*	*y*	*z*	*U*_iso_*/*U*_eq_	
Cu	0.09830 (10)	0.76976 (9)	0.14071 (7)	0.0155 (2)	
N1	−0.0741 (6)	0.7152 (5)	0.2890 (4)	0.0185 (11)	
C2	−0.0718 (8)	0.7285 (7)	0.4124 (5)	0.0220 (13)	
H2	0.0206	0.7691	0.4361	0.026*	
C3	−0.1984 (8)	0.6857 (7)	0.5071 (6)	0.0247 (14)	
H3	−0.1926	0.6958	0.5939	0.030*	
C4	−0.3340 (8)	0.6274 (7)	0.4709 (6)	0.0263 (14)	
H4	−0.4235	0.5976	0.5331	0.032*	
C5	−0.3374 (8)	0.6133 (7)	0.3427 (5)	0.0244 (14)	
H5	−0.4291	0.5738	0.3167	0.029*	
C6	−0.2058 (7)	0.6573 (6)	0.2541 (5)	0.0169 (12)	
C7	−0.2052 (8)	0.6497 (6)	0.1132 (5)	0.0180 (12)	
O7	−0.3162 (5)	0.5945 (5)	0.0733 (4)	0.0232 (9)	
N8	−0.0714 (6)	0.7116 (5)	0.0432 (4)	0.0164 (10)	
C9	−0.0375 (7)	0.7206 (6)	−0.0861 (5)	0.0151 (12)	
O9	−0.1195 (5)	0.6830 (5)	−0.1644 (4)	0.0227 (9)	
C10	0.1237 (7)	0.7882 (6)	−0.1305 (5)	0.0163 (12)	
C11	0.1863 (8)	0.8111 (6)	−0.2593 (5)	0.0195 (13)	
H11	0.1276	0.7848	−0.3234	0.023*	
C12	0.3370 (8)	0.8732 (7)	−0.2914 (5)	0.0237 (13)	
H12	0.3830	0.8901	−0.3783	0.028*	
C13	0.4194 (8)	0.9104 (7)	−0.1954 (5)	0.0228 (13)	
H13	0.5227	0.9528	−0.2159	0.027*	
C14	0.3502 (8)	0.8853 (7)	−0.0697 (6)	0.0229 (13)	
H14	0.4076	0.9103	−0.0041	0.028*	
N15	0.2036 (6)	0.8264 (5)	−0.0380 (4)	0.0165 (10)	
N16	0.2400 (7)	0.8814 (6)	0.2117 (5)	0.0241 (12)	
N17	0.2333 (6)	0.9146 (6)	0.3164 (5)	0.0206 (11)	
N18	0.2343 (8)	0.9525 (7)	0.4143 (5)	0.0365 (14)	
O1	0.3387 (5)	0.5191 (5)	0.1809 (4)	0.0217 (9)	
H1A	0.305 (8)	0.455 (6)	0.147 (5)	0.026*	
H1B	0.440 (5)	0.520 (7)	0.138 (5)	0.026*	

### Atomic displacement parameters (Å^2^)

**Table:**

	*U*^11^	*U*^22^	*U*^33^	*U*^12^	*U*^13^	*U*^23^
Cu	0.0162 (4)	0.0193 (4)	0.0141 (4)	−0.0097 (3)	0.0013 (3)	−0.0040 (3)
N1	0.018 (3)	0.023 (3)	0.016 (3)	−0.008 (2)	−0.001 (2)	−0.004 (2)
C2	0.023 (3)	0.029 (3)	0.020 (3)	−0.015 (3)	0.004 (3)	−0.010 (3)
C3	0.033 (3)	0.029 (4)	0.015 (3)	−0.013 (3)	0.007 (3)	−0.007 (3)
C4	0.027 (3)	0.029 (4)	0.023 (3)	−0.014 (3)	0.008 (3)	−0.004 (3)
C5	0.022 (3)	0.031 (4)	0.027 (3)	−0.016 (3)	0.005 (3)	−0.013 (3)
C6	0.020 (3)	0.010 (3)	0.018 (3)	−0.003 (2)	0.002 (2)	−0.002 (2)
C7	0.020 (3)	0.013 (3)	0.021 (3)	−0.005 (2)	0.000 (2)	−0.006 (2)
O7	0.023 (2)	0.028 (2)	0.026 (2)	−0.0158 (19)	0.0003 (18)	−0.0074 (18)
N8	0.019 (2)	0.019 (3)	0.016 (3)	−0.012 (2)	0.000 (2)	−0.004 (2)
C9	0.016 (3)	0.014 (3)	0.015 (3)	−0.005 (2)	−0.006 (2)	0.001 (2)
O9	0.021 (2)	0.028 (2)	0.021 (2)	−0.0119 (18)	−0.0018 (18)	−0.0023 (18)
C10	0.016 (3)	0.013 (3)	0.019 (3)	−0.005 (2)	0.000 (2)	−0.001 (2)
C11	0.021 (3)	0.022 (3)	0.015 (3)	−0.007 (3)	−0.004 (2)	0.000 (2)
C12	0.024 (3)	0.029 (3)	0.016 (3)	−0.008 (3)	0.005 (2)	−0.002 (3)
C13	0.016 (3)	0.028 (3)	0.025 (3)	−0.012 (3)	0.006 (2)	0.000 (3)
C14	0.020 (3)	0.020 (3)	0.032 (4)	−0.011 (3)	0.001 (3)	−0.005 (3)
N15	0.012 (2)	0.020 (3)	0.019 (3)	−0.007 (2)	−0.0003 (19)	−0.003 (2)
N16	0.026 (3)	0.034 (3)	0.022 (3)	−0.022 (2)	0.001 (2)	−0.009 (2)
N17	0.022 (3)	0.024 (3)	0.020 (3)	−0.014 (2)	−0.005 (2)	−0.001 (2)
N18	0.044 (3)	0.052 (4)	0.024 (3)	−0.026 (3)	−0.004 (3)	−0.011 (3)
O1	0.016 (2)	0.025 (2)	0.025 (2)	−0.0093 (18)	0.0014 (17)	−0.0069 (18)

### Geometric parameters (Å, º)

**Table:**

Cu—N16	1.958 (4)	N8—C9	1.359 (7)
Cu—N8	1.961 (4)	C9—O9	1.235 (6)
Cu—N15	2.007 (4)	C9—C10	1.505 (7)
Cu—N1	2.009 (4)	C10—N15	1.350 (7)
Cu—O1	2.319 (4)	C10—C11	1.393 (7)
N1—C2	1.341 (6)	C11—C12	1.390 (7)
N1—C6	1.359 (6)	C11—H11	0.9500
C2—C3	1.388 (7)	C12—C13	1.386 (8)
C2—H2	0.9500	C12—H12	0.9500
C3—C4	1.393 (8)	C13—C14	1.380 (8)
C3—H3	0.9500	C13—H13	0.9500
C4—C5	1.394 (8)	C14—N15	1.344 (6)
C4—H4	0.9500	C14—H14	0.9500
C5—C6	1.382 (7)	N16—N17	1.198 (6)
C5—H5	0.9500	N17—N18	1.157 (6)
C6—C7	1.509 (7)	O1—H1A	0.85 (2)
C7—O7	1.236 (6)	O1—H1B	0.84 (2)
C7—N8	1.375 (7)		
			
N16—Cu—N8	165.67 (19)	N8—C7—C6	111.0 (4)
N16—Cu—N15	91.65 (19)	C9—N8—C7	124.6 (4)
N8—Cu—N15	80.91 (18)	C9—N8—Cu	118.2 (3)
N16—Cu—N1	103.67 (19)	C7—N8—Cu	116.9 (3)
N8—Cu—N1	82.26 (18)	O9—C9—N8	129.1 (5)
N15—Cu—N1	162.45 (16)	O9—C9—C10	120.3 (5)
N16—Cu—O1	93.76 (18)	N8—C9—C10	110.7 (4)
N8—Cu—O1	98.80 (16)	N15—C10—C11	121.8 (5)
N15—Cu—O1	92.89 (16)	N15—C10—C9	115.9 (4)
N1—Cu—O1	94.57 (16)	C11—C10—C9	122.3 (5)
C2—N1—C6	119.0 (5)	C12—C11—C10	118.3 (5)
C2—N1—Cu	128.2 (4)	C12—C11—H11	120.8
C6—N1—Cu	112.8 (3)	C10—C11—H11	120.8
N1—C2—C3	122.9 (5)	C13—C12—C11	119.4 (5)
N1—C2—H2	118.5	C13—C12—H12	120.3
C3—C2—H2	118.5	C11—C12—H12	120.3
C2—C3—C4	118.0 (5)	C14—C13—C12	119.4 (5)
C2—C3—H3	121.0	C14—C13—H13	120.3
C4—C3—H3	121.0	C12—C13—H13	120.3
C3—C4—C5	119.4 (5)	N15—C14—C13	121.6 (5)
C3—C4—H4	120.3	N15—C14—H14	119.2
C5—C4—H4	120.3	C13—C14—H14	119.2
C6—C5—C4	119.3 (5)	C14—N15—C10	119.5 (5)
C6—C5—H5	120.4	C14—N15—Cu	126.3 (4)
C4—C5—H5	120.4	C10—N15—Cu	114.0 (3)
N1—C6—C5	121.4 (5)	N17—N16—Cu	131.4 (4)
N1—C6—C7	116.6 (4)	N18—N17—N16	175.6 (5)
C5—C6—C7	122.0 (5)	Cu—O1—H1A	106 (4)
O7—C7—N8	127.9 (5)	Cu—O1—H1B	112 (4)
O7—C7—C6	121.1 (5)	H1A—O1—H1B	101 (5)
			
N16—Cu—N1—C2	−9.4 (5)	O1—Cu—N8—C7	87.4 (4)
N8—Cu—N1—C2	−176.2 (5)	C7—N8—C9—O9	2.8 (9)
N15—Cu—N1—C2	−159.6 (5)	Cu—N8—C9—O9	176.3 (4)
O1—Cu—N1—C2	85.6 (5)	C7—N8—C9—C10	−177.7 (5)
N16—Cu—N1—C6	171.0 (4)	Cu—N8—C9—C10	−4.1 (6)
N8—Cu—N1—C6	4.3 (4)	O9—C9—C10—N15	179.7 (5)
N15—Cu—N1—C6	20.9 (8)	N8—C9—C10—N15	0.1 (6)
O1—Cu—N1—C6	−94.0 (4)	O9—C9—C10—C11	0.0 (8)
C6—N1—C2—C3	0.0 (8)	N8—C9—C10—C11	−179.6 (5)
Cu—N1—C2—C3	−179.5 (4)	N15—C10—C11—C12	0.8 (8)
N1—C2—C3—C4	−0.4 (9)	C9—C10—C11—C12	−179.6 (5)
C2—C3—C4—C5	0.4 (9)	C10—C11—C12—C13	0.0 (8)
C3—C4—C5—C6	0.0 (9)	C11—C12—C13—C14	−0.1 (8)
C2—N1—C6—C5	0.5 (8)	C12—C13—C14—N15	−0.4 (8)
Cu—N1—C6—C5	−179.9 (4)	C13—C14—N15—C10	1.1 (8)
C2—N1—C6—C7	178.3 (5)	C13—C14—N15—Cu	175.7 (4)
Cu—N1—C6—C7	−2.1 (6)	C11—C10—N15—C14	−1.3 (8)
C4—C5—C6—N1	−0.5 (8)	C9—C10—N15—C14	179.1 (5)
C4—C5—C6—C7	−178.2 (5)	C11—C10—N15—Cu	−176.6 (4)
N1—C6—C7—O7	177.9 (5)	C9—C10—N15—Cu	3.8 (6)
C5—C6—C7—O7	−4.3 (8)	N16—Cu—N15—C14	12.8 (5)
N1—C6—C7—N8	−2.6 (7)	N8—Cu—N15—C14	−179.5 (5)
C5—C6—C7—N8	175.2 (5)	N1—Cu—N15—C14	163.8 (5)
O7—C7—N8—C9	−0.7 (9)	O1—Cu—N15—C14	−81.1 (4)
C6—C7—N8—C9	179.9 (5)	N16—Cu—N15—C10	−172.3 (4)
O7—C7—N8—Cu	−174.3 (4)	N8—Cu—N15—C10	−4.6 (4)
C6—C7—N8—Cu	6.3 (6)	N1—Cu—N15—C10	−21.3 (8)
N16—Cu—N8—C9	64.4 (10)	O1—Cu—N15—C10	93.8 (4)
N15—Cu—N8—C9	4.9 (4)	N8—Cu—N16—N17	112.7 (8)
N1—Cu—N8—C9	179.9 (4)	N15—Cu—N16—N17	171.0 (5)
O1—Cu—N8—C9	−86.6 (4)	N1—Cu—N16—N17	−0.4 (6)
N16—Cu—N8—C7	−121.6 (8)	O1—Cu—N16—N17	−96.0 (5)
N15—Cu—N8—C7	179.0 (4)	Cu—N16—N17—N18	−171 (7)
N1—Cu—N8—C7	−6.0 (4)		

### Hydrogen-bond geometry (Å, º)

**Table:**

*D*—H···*A*	*D*—H	H···*A*	*D*···*A*	*D*—H···*A*
O1—H1*A*···O9^i^	0.85 (2)	2.10 (4)	2.843 (5)	145 (5)
O1—H1*B*···O7^ii^	0.84 (2)	2.13 (3)	2.922 (5)	157 (5)
O1—H1*A*···O7^i^	0.85 (2)	2.45 (4)	3.105 (5)	134 (5)

Symmetry codes: (i) −*x*, −*y*+1, −*z*; (ii) *x*+1, *y*, *z*.
